# Heat Transfer Analysis of Warm Guss Asphalt Concrete for Mini-Trench Overlaying

**DOI:** 10.3390/ma16072808

**Published:** 2023-03-31

**Authors:** Kyung-Nam Kim, Yeong-Min Kim, Sang-Yum Lee, Tri Ho Minh Le

**Affiliations:** 1 Korea Institute of Civil Engineering and Building Technology, 283 Goyangdae-Ro, Ilsanseo-Gu, Goyang-si 10223, Republic of Korea; kimkyungnam@kict.re.kr (K.-N.K.); choozang@kict.re.kr (Y.-M.K.); 2 Faculty of Civil Engineering, Induk University, 12 Choansan-ro Nowon-gu, Seoul 01878, Republic of Korea; 3 Faculty of Civil Engineering, Nguyen Tat Thanh University, 300A Nguyen Tat Thanh Street, District 4, Ho Chi Minh City 70000, Vietnam; lhmtri@ntt.edu.vn

**Keywords:** heat transfer analysis, warm guss asphalt, overlaying asphalt concrete, buried conduit, backfill concrete, rapid construction

## Abstract

Conventional hot mix asphalt overlaying on trench infrastructure typically necessitates extended cooling times for further works and can have adverse effects on buried components, such as electricity cables and hot water pipes. Therefore, this research aims to investigate the use of warm guss mastic asphalt (at an installation temperature of 160 °C) as an overlaying material for mini-trenches, which can reduce the cooling time required for traffic opening and improve the efficiency of the construction process. This research involved two stages: first, lab testing and related research results were used to generate the thermal conductivity and specific heat necessary for simulation work. Second, a finite element model analysis was conducted to evaluate the thermal transmission of the overlaying surface and the buried conduit based on the summer pavement temperature distribution through the Korean Pavement Research Program. Afterward, the field test bed was constructed to verify the simulation. The results indicate that the optimal thickness of the overlaying material and the concrete covering should be designed to ensure thermal durability and meet traffic opening requirements. The overlaying depth of the mini trench using warm mix guss mastic asphalt should be less than 100 mm to meet with the traffic opening time, while the thickness of the concrete covering should be designed to be more than 100 mm to ensure thermal durability. Additionally, the findings suggest that the application of warm guss asphalt could reduce the opening time by 30 min to 1 h and 25 min compared to conventional hot guss asphalt materials. When the pavement surface temperature for the traffic opening is controlled at 50 °C, the asphalt mixture requires at least 2 h to 5 h to meet the cooling criteria for traffic opening, respectively. Overall, this research confirms the potential benefits and optimal use of warm guss mastic asphalt in the construction process of mini-trenches.

## 1. Introduction

With the development of new communication technologies such as high-speed internet, internet protocol television, cable TV, broadcasting, communication service providers have been competing to install application cables. As a result, the aerial cables in the downtown area are crowded, impairing the urban aesthetics, threatening traffic safety with the installation of aerial cables that do not meet regulations, and increasing the risk of accidents [1]. Therefore, demand for undergrounding new and existing communication cables is continuously increasing due to the problem of overcrowding of aerial cables, disaster prevention, and the improvement of urban aesthetics [2,3,4].

In accordance with the Comprehensive Aerial Maintenance Plan (November 2012), the Korean project for undergrounding aerial lines was promoted with the budget of investing a total of 1.5 trillion Korea won for 5 years (2013–2017). After 4 years of the project, a study was conducted on the development of a new underground aerial burial method and management technology for the regeneration of old cities, and a domestic application standard for the mini-trenching method was introduced [5]. However, until now, Korean national standards for the mini-trenching method have not been clearly established due to the thermal concerns induced by the overlaying asphalt to the conduit. As a result, there is a crucial need for an alternative that can reduce construction resources with advanced technology.

Guss mastic asphalt mixtures are impermeable asphalt mixtures produced at a temperature of about 240 °C [6]. Its general application was practical for bridge pavement, but recently it has been used for earthwork pavement because of its excellent adhesion, imperviousness, and high durability [7]. Guss asphalt has high fluidity and is characterized by self-leveling by its weight without the need for separate compaction [8]. These characteristics enable the use of asphalt mixtures in locations where compaction is not possible, and in particular, it can be better applied to narrow areas where compaction is difficult, such as mini-trenching [6,9]. Guss asphalt mixtures have significant limitations in their use despite these excellent properties because the production temperature is approximately 240 °C and the compaction is performed at a high temperature of about 200 °C [10,11,12]. In order to overcome these limitations, technology has been recently developed to produce guss asphalt by applying medium-temperature additives [6,13,14] to lower the production and construction temperatures by about 20 °C to 40 °C compared to the existing ones [9,15]. Additionally, the control of the proper mix design and temperature of the asphalt concrete mixture can further contribute to the stronger foundation of the infrastructure system [16,17,18], promoting the application of modified HMA for multiple purposes.

Currently, there is a lack of research focus on the practical application of guss mastic asphalt as an overlaying material for buried conduit due to the major concern of overheating issues. Moreover, studies on the thermal transmission from the guss mastic asphalt to the buried conduit were limited. Therefore, this research aims to evaluate the potential application of this material through a simulation process. This research focuses on the practical application of warm guss mastic asphalt as an alternative overlaying material for mini-trenches to reduce the cooling time required for traffic opening and improve the efficiency of the construction process. Throughout the laboratory and simulation works, the generated results are expected to suggest an optimal thickness for the overlaying material and concrete covering to ensure thermal durability and meet traffic opening requirements, thereby contributing to the accelerated construction process and minimizing energy consumption on the planet.

**Figure 1:**
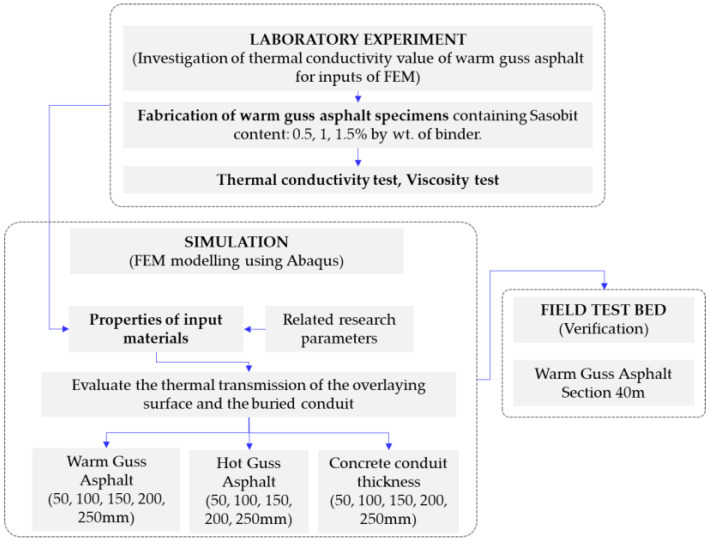
Research flowchart.

In this study, a series of laboratory tests were conducted to initially obtain the thermal conductivity of the proposed guss mastic asphalt for simulation works. In order to develop a practical solution using warm guss asphalt (WGA) as an overlaying material for rapid mini-trench construction, the heat transfer analysis was performed using the finite element method. The temperature from the heat of the guss asphalt mixture was designed to transfer through the interlayer protective concrete surrounding the buried pipe, and the surface of the buried pipe was evaluated following the Korean criteria, which meet the proper operation temperature of about 50 °C. In addition, the surface temperature of the guss asphalt mixture laid in the trench and the traffic opening time following the maintenance standards [19] were investigated. In addition, the conventional hot guss asphalt (HGA) with a compaction temperature of 200 °C was also analyzed and compared with the newly developed WGA. Finally, the full-scale testbed was constructed to verify the accuracy of the simulation model. The summary of the testing process is presented in Figure 1.

## 2. Materials and Methods

### 2.1. Laboratory Testing

#### 2.1.1. Modified Asphalt Binder

In this study, styrene-butadiene-styrene (SBS) modified asphalt binder was utilized [20]. SBS is well known for its ability to improve the elasticity of a binder at high temperatures and its adaptability at cold temperatures [11,13,21]. In order to increase the compatibility of guss mastic asphalt, this SBS modifier can strengthen the high-temperature performance of an asphalt mix by lowering the mixing temperature and the viscosity of high-temperature asphalt [1,20,22]. The proposed bitumen exhibits greater resilience to low-temperature cracking and also to rutting at greater temperatures than normal asphalt. This material has a long history of successful application in the Republic of Korea [9].

#### 2.1.2. Binder Modified by Sasobit

The white spherical Sasobit additive used in this research has a density of 0.88 g/cm^3^ at 25 °C, and a melting point of 81.5 °C, and a flash point of 280 °C [11,13]. Regarding the binder mixing process, an adequate quantity of asphalt was preheated in the mixer equipment to 160 °C and it was then mixed for 30 min. Afterward, the proper Sasobit contents were then applied, and the combination was stirred for an additional 15 min to create the initial form of guss asphalt mixture. Consequently, a mixer stirred the combined asphalt binder for 30 min at a speed of 3000 rpm while keeping the mixing temperature at 160 °C to produce the homogenous binder [11,12,13,21].

The rolling thin film oven test (RTFO) was employed to age the binders for 75 min at 160 °C to simulate the aging process at a warmer temperature. This approach was performed at 160 °C instead of 180 °C although gusss mastic asphalt mixtures are often manufactured in asphalt plants at a higher mixing temperature than conventional asphalt concrete mixtures. Table 1 presents the general properties of guss mastic asphalt modified with 1% Sasobit by weight of the binder.

**Figure 2:**
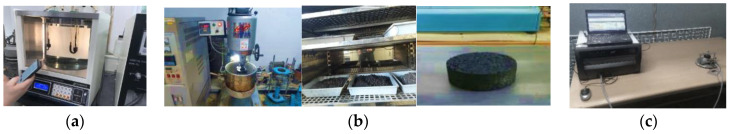
Illustration of laboratory test. (**a**) absolute viscosity; (**b**) Test specimen fabrication of Guss mastic asphalt mixtures; (**c**) Thermal conductivity test.

In this research, the natural aggregate and fillers were provided by the Korean Plant near the Seoul city area. The general properties of aggregate and gradation were presented in Table 2, and Table 3, respectively. Considering the fabrication of a guss mastic asphalt mixture for the thermal conductivity test, the proposed research produced specimens in accordance with the Superpave compaction method. Based on the trial experiments and suggestions from related research [12,14,21], the optimized asphalt binder is 10.75% by weight of the mixture. Regards to the development of test specimens, this process involves the determination of the viscosity of the generated warm mix binder containing Sasobit (see Figure 2a). Afterward, the specimens were fabricated based on the Superpave mix design as shown in Figure 2b. Consequently, Figure 2c depicts the thermal conductivity performed to quantify the input value for the simulation process.

The fabricated specimens were designed to have a thickness of 20 cm and a diameter of 10cm for the thermal conductivity test as depicted in Figure 2b [23]. In this research, the primary focus is the heat transfer analysis for the development of practical WGA for overlaying purposes. Therefore, only some general properties of specimens were investigated (flow value and stability test). The overall strength test result is shown in Table 4.

#### 2.1.3. Thermal Conductivity Test

The thermal conductivity of various Sasobit content in guss asphalt mixtures was investigated to provide an input property for the simulation process. By employing a Thermal Constants Analyzer (Figure 2c), examinations on cylindrical specimens having a height of 20 mm and diameter of 10 mm were performed. This device could determine thermal conductivity between 0.01 and 600 W/mK. The procedure complied with ISO Standard 22,007-2 [24] and it was calibrated to meet the standard accuracy [23]. To assess their thermal characteristics, a sensor was positioned in between two samples. Regarding the average result, one mix condition was represented by three replications.

### 2.2. Heat Transfer Analysis

In this section, the theory of heat transfer, finite element analysis, model construction, physical properties, initial conditions, boundary conditions, and analysis methods are respectively described. ABAQUS, a finite element analysis program, was used to analyze the effect of the heat of the guss asphalt mixture on the temperature change of the pavement and buried pipe [2,25,26].

#### 2.2.1. Finite element Method of Heat Transfer

##### Heat Conduction Governing Equation

Based on the first law of thermodynamics that thermal energy is conserved, the conservation of thermal energy in the numerical domain can be expressed as follows [25,26,27].

(1) $$\rho C\frac{\partial T}{\partial t}+{\left\{L\right\}}^{T}\left\{q\right\}=0$$

where, ρ is the density (kg/m^3^), *C* is specific heat (J/kgK), T is the temperature at each location over time, *t* is the time, L=∂∂x∂∂y∂∂z is a vector operator, and $\left\{q\right\}$ is the heat flux vector.

In Equation (1) above, ${\left\{L\right\}}^{T}\left\{q\right\}$ can be interpreted as ∇ ⋯ $\left\{q\right\}$ and ∇ is the divergence operator. According to Fourier’s law of heat conduction [25,26,27], the heat flux vector can be expressed in relation to the temperature gradient, as shown in Equation (2) below.

(2) $$\left\{q\right\}=-\left[D\right]\left\{L\right\}T$$

where, $\left[D\right]=\left[\begin{array}{ccc} K_{xx} & 0 & 0\\ 0 & K_{yy} & 0\\ 0 & 0 & K_{zz} \end{array}\right]$ is the thermal conductivity matrix. $K_{xx}$, $K_{yy}$, and $K_{zz}$ are the thermal conductivity in each direction.

If the above expression is expanded and rearranged, it can be expressed as the following Equation (3). By assuming an isotropic material ($K=K_{xx}=K_{yy}=K_{zz}$), it can be finally expressed as the following Equation (4).

(3) $$\rho C\frac{\partial T}{\partial t}=\frac{\partial }{\partial x}\left(K_{xx}\frac{\partial T}{\partial x}\right)+\frac{\partial }{\partial x}\left(K_{yy}\frac{\partial T}{\partial y}\right)+\frac{\partial }{\partial x}\left(K_{zz}\frac{\partial T}{\partial z}\right)$$

(4) $$\rho C\frac{\partial T}{\partial t}=\frac{\partial }{\partial x}K\left\{\left(\frac{\partial T}{\partial x}\right)+\frac{\partial }{\partial x}\left(\frac{\partial T}{\partial y}\right)+\frac{\partial }{\partial x}\left(\frac{\partial T}{\partial z}\right)\right\}$$

#### 2.2.2. Boundary Conditions

The boundary conditions for heat transfer analysis are generally considered to be of three types. The heat that directly enters the model surface, such as solar radiation, convection from the model surface, and radiant heat emitted from the model surface to the outside [25,26,27]. The heat flow directly to the surface can be expressed as Equation (5) below.

(5) $${\left\{q\right\}}^{T}\left\{\eta \right\}=-q^{*}$$

where {η} is the unit normal vector in the direction outside the model, q* is the heat flow coming from the outside (solar radiation, etc.). The heat flow acting on the surface by convection is given by Equation (6) below.

(6) $${\left\{q\right\}}^{T}\left\{\eta \right\}=h_{f}\left(T_{surf}-T_{air}\right)$$

where $h_{f}$ is the convection coefficient, $T_{surf}$ is the surface temperature, $T_{air\;}$ is the air temperature [25]. The radiant energy exchange between the surface of the model and its surroundings is

(7) $$q_{c}=\varepsilon \sigma \left(T_{surf}^{4}-T_{air}^{4}\right)$$

where $q_{c\;}$ long-wave radiant intensity balance, ε is the effective emissivity, σ is the Stefan Boltzmann constant.

### 2.3. Composition and Elements of Analytical Model

**Figure 3:**
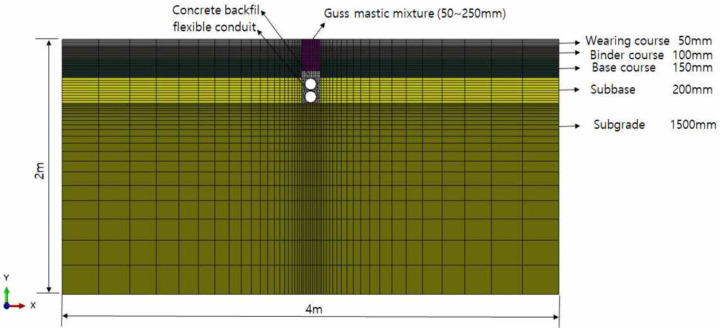
Finite Element Model for Heat Transfer Analysis.

The finite element analysis model for pavement analysis was constructed in two dimensions for simple analysis, while each pavement layer and buried pipe were assumed to be a continuum [25,26]. The size of the analytical model is designed based on the suggestions from the Korean standard [5,28] and related research [25]. As shown in Figure 3, the size of the model was set to 4 m in length and 2 m in depth so that the heat transmitted by the heat source was trapped and did not affect the analysis [25,29]. Following the requirements in practice, the pavement structure was designed to have a thickness of 50 mm for the surface layer, 100 mm for the middle layer, 150 mm for the base layer, 200 mm for the subbase layer, and 1500 mm for the subgrade layer [30].

In this research, a DC2D4 heat transfer element with four nodes was applied as the element used in the heat transfer analysis, and the size of one element was densely meshed with an average size of about 10 mm around the guss asphalt mixture, which is the primary heat source [25]. Referring to a previous research project [5] for the mini-trench as shown in the following Figure 3, a flexible buried pipe having a thickness of 5 mm and an outer diameter of 100 mm was buried in a trench having a width of 150 mm and a depth of 500 mm. In addition, the surrounding material of the buried pipe is composed of ultra-fast setting backfill concrete for the protection of the buried pipe [29], and the guss asphalt mixture is overlayed on top of it. As presented in Table 5, the analysis was performed by varying the thickness of the guss mastic asphalt layer from 50 to 250 mm, while the initial transmitting temperatures of warm guss and normal guss asphalt were set at 160 °C and 200 °C, respectively.

### 2.4. Physical Properties of Materials

Based on the literature review of related studies and suggestions from prior research, the density, thermal conductivity, and specific heat of each material for heat transfer analysis were determined as shown in Table 6. Luca and Mrawira [31] performed thermal properties tests on asphalt mixtures with densities ranging from 2297 kg/m^3^ to 2450 kg/m^3^, and the generated thermal conductivity ranged from 1.6 W/mK to 2.1 W/mK. Besides, the specific heat of the material ranged from 1475 J/kgK to 1853 J/kgK. Considering the granular material, Côté et al. [32] tested the thermal conductivity of granular material with a general density of 1545 kg/m^3^ to 2405 kg/m^3^, and the result was distributed from 1.8 W/mK to 3.2 W/mK. Regarding the granular layer, Ižvolt et al. tested the specific heat of the granular material, and the extracted results ranged from 918 J/kgK to 1,091 J/kgK [33,34]. Regarding the subgrade layer, Xu et al. [35] showed that the thermal conductivity of subgrade materials varied from 0.7 W/mK to 2.2 W/mK under various moisture content and temperature conditions of the subgrade materials (densities ranging from 1769 kg/m^3^ to 2027 kg/m^3^). Finally, Kay et al. [36] investigated the specific heat of the subbase material, and the results show the result was distributed from 875 J/kgK to 1968 J/kgK. Besides, according to ACI-122R-02, the specific heat of concrete backfill is 838 J/kgK~1088 J/kgK.

In addition, the thermal conductivity can be determined by the following Equation (8) for fully dried concrete with a density in the range of 240 kg/m^3^ to 2400 kg/m^3^, and a value 1.25 times larger can be assumed in the wet state. The flexible buried pipe was assumed to be made of PVC material, and the density was determined to be 1380 kg/m^3^, the thermal conductivity to be 0.2 W/mK, and the specific heat to be 880 J/kgK [28].

(8) $$k_{c}=0.072\exp\left(0.00125d\right)$$

where *k_c_* is thermal conductivity as a function of the perfect dry density of concrete (W/mK) and *d* is the perfect dry density (kg/m^3^).

### 2.5. Initial and Boundary Conditions

#### 2.5.1. Initial Conditions

**Figure 4:**
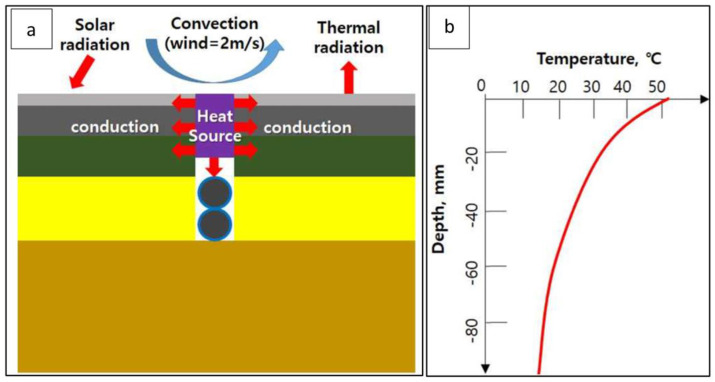
Initial and boundary conditions. (**a**) The transmitting of heat source; (**b**) the relationship between temperature and depth.

Figure 4a conceptually shows the initial and boundary conditions considered in the heat transfer analysis of this study. As for the initial conditions, the environment with the highest temperature of the pavement surface in the summer of the Republic of Korea was considered since this is the worst condition for the temperature of the high-temperature guss asphalt [25]. The initial temperature of the pavement, as shown in Figure 4b, was determined. The temperature at each depth was calculated using the Korean Pavement Design Method temperature analysis program under the condition of 2:00 pm in July in Seoul [19] (the time when the temperature of the surface layer is the highest).

#### 2.5.2. Thermal Load Case

As the primary heat source from the overlaying material, the heat load condition was defined to move the heat source by setting the temperature of the guss asphalt to 160 °C for the WGA mixture and 200 °C for the conventional guss asphalt admixture.

#### 2.5.3. Boundary Conditions

Referring to the Korea Meteorological Administration data, it was assumed that 100 W/m^2^ was applied to the external inflow of radiant heat by solar radiation. Considering the convective boundary conditions shown in Equation (6), the convection coefficient model of Branco et al. [38] was applied as shown in Equation (9) with the assumed air temperature of 30 °C and the wind speed of 2 m/s. In addition, based on Equation (7) and the atmospheric radiation boundary condition, the effective emission coefficient (ε) of asphalt was determined to be 0.93 and the Stefan Boltzmann constant was 5.67 × 10^−8^ W/m^2^K^4^.

(9) $$h_{c}=6+3.7v_{\omega },\;(W/m^{2}/{}^{\circ}C)$$

where, $v_{\omega }$ is the wind speed (m/s).

#### 2.5.4. Analysis Method

The temperature of the pavement and buried pipe over time was analyzed in the initial and boundary conditions [25]. In this analysis, the implicit finite element method was employed, and the analysis time was given a total of 24 h while Table 7 summarizes the criteria for evaluation ([28]). The analysis time increment was set to 10 min and analysis results for a total of 144 increments were obtained.

**Figure 5:**
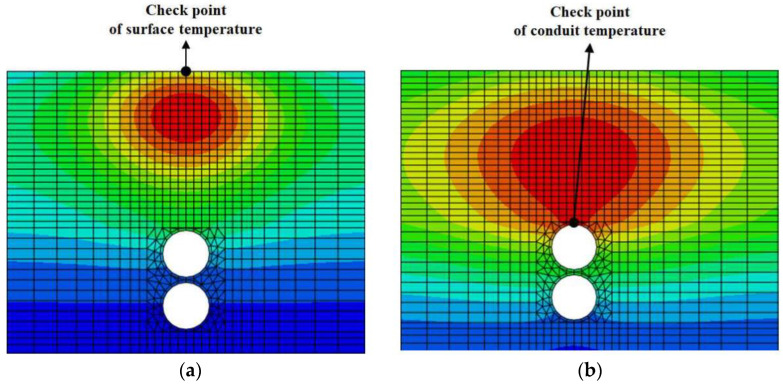
Checkpoint of FEA results. (**a**) Pavement surface temperature; (**b**) Conduit temperature.

As shown in Figure 5a, the temperature at the surface of the overlaying was checked at each increment to investigate whether the overlaying of the guss asphalt mixture fell below the traffic opening temperature in a required interval. In addition, as shown in Figure 5b, the temperature was also recorded at each time increment at the top of the pipe, which is closest to the heat source, to check whether the buried pipe rose above the operating temperature [29,39].

## 3. Results

### 3.1. Thermal Conductivity Test Results

**Figure 6:**
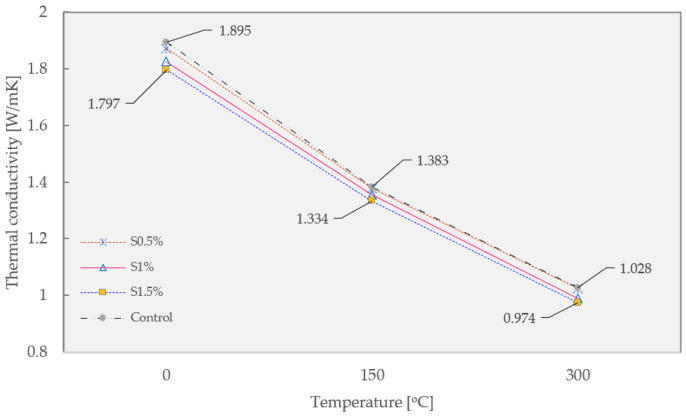
Thermal conductivity test results of guss mastic asphalt mixture modified by Sasobit.

The thermal conductivity of guss mastic asphalt modified by different Sasobit content is presented in Figure 6 below. In general, the application of Sasobit slightly impacts the thermal conductivity of guss mastic asphalt. It may be attributed to the replacement of mastic asphalt binder by Sasobit, which obtains lower thermal resistivity compared to the former component. The effect of this additive is neglectable since the highest Sasobit content of 1.5% results in a thermal conductivity drop of 5.17, 3.54, and 5.25% compared to the control mixture at 0, 150, and 300 °C, respectively. Therefore, the Sasobit content is controlled at 1% to minimize the side impact on the guss mastic asphalt.

**Figure 7:**
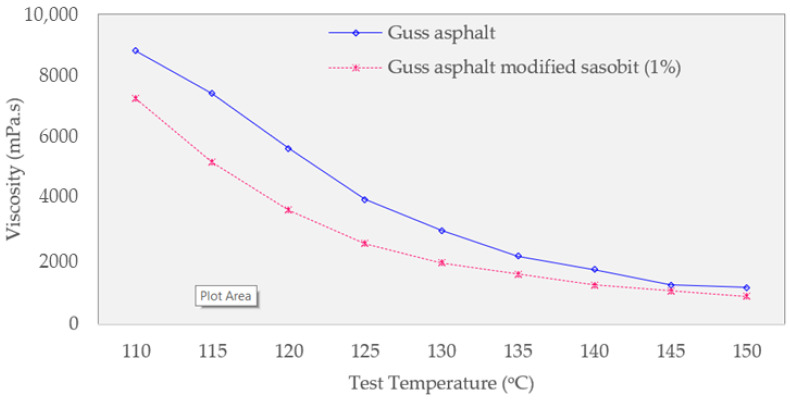
Viscosity analysis of guss asphalt mixtures.

In all test temperatures, the viscosity of the Sasobit-modified asphalt is lower than that of the original asphalt, as shown in Figure 7. This finding suggests that the Sasobit additive can successfully reduce the viscosity of the guss asphalt. The test temperature of the Sasobit mixture can be reduced by roughly 7 °C at the equivalent viscosity compared to the control guss mastic asphalt, suggesting the potential for construction effectiveness.

### 3.2. Overlaying Surface Temperature

Korean asphalt mixture production and construction guidelines [19] require that traffic can be opened when the surface temperature of the pavement is less than 40 °C after asphalt maintenance (or overlaying), and traffic can even be operated at less than 50 °C when the supervisor’s approval is also accepted in some areas. Therefore, based on this requirement, the analysis results of WGA overlaying and HGA overlaying were examined.

**Figure 8:**
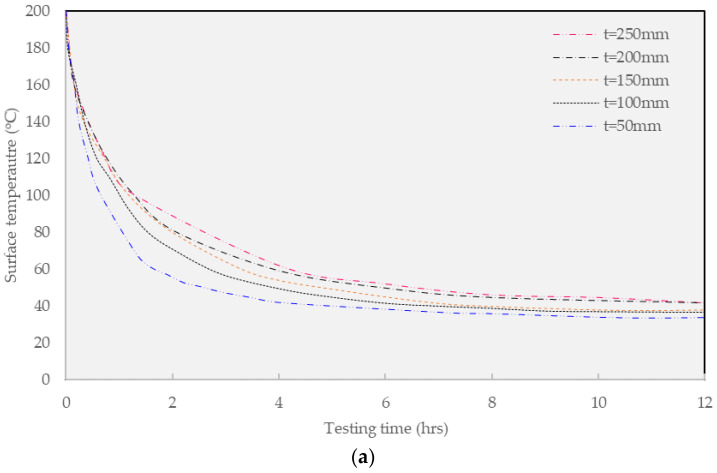

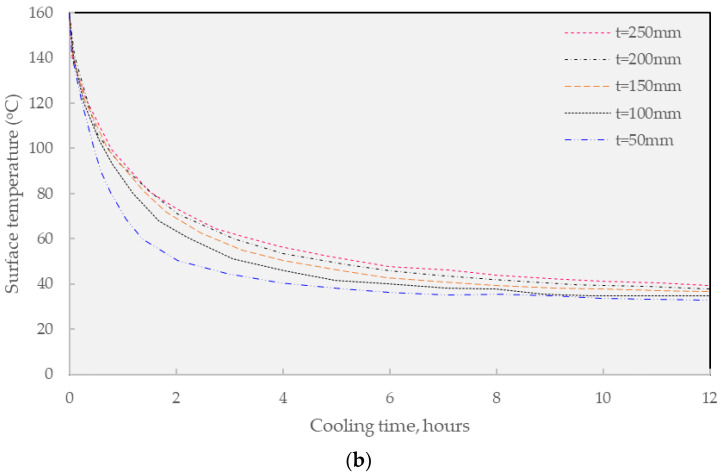
Pavements surface temperatures of FEA results. (**a**) Hot Mix Guss Mastic Asphalt; (**b**) Warm Mix Guss Mastic Asphalt.

Figure 8 shows the impact of the thickness of the overlaying material (from t = 50 mm to t = 250 mm) and the type of overlaying material (warm and hot guss asphalt) on thermal dissipation for about 12 h after overlaying construction. It shows the analysis results of the temperature change on the pavement surface. In addition, the traffic opening standards based on the guidelines of the Ministry of Land, Infrastructure, and Transport are presented as the correlated red line.

Figure 8a depicts the findings from the simulation of general HGA overlaying (200 °C). In general, the thickness of warm mix asphalt concrete noticeably contributes to the surface temperature of the layer. The higher thickness results in a greater required cooling time for traffic opening. When the thickness of the overlaying is 50 mm, the traffic opening time based on 40 °C criteria is 4 h 40 min after overlaying, while this value of 50 °C condition can be achieved after 2 h and 30 min. Further, this value of the 100 mm guss mastic asphalt layer concept is 7 h and 4 h in the former and latter constraints, respectively. Regarding the largest overlaying thickness of 250 mm, the required traffic opening time at 40 °C is up to 12 h 45 min, while the traffic opening time of the greater temperature 50 °C is 6 h and 40 min. This finding confirms the noticeable impact of thickness on the constituted thermal energy. Further investigation was performed on the warm mix asphalt.

Figure 8b summarizes the result of the WGA overlaying concept (160 °C). In general, the findings share the equivalent trend as found in the HGA mixture since the shortened asphalt layer thickness greatly cultivates the drop in the required cooling time, especially, at t = 50mm. Considering the effectiveness of WGA, the results suggest that the application of this technique may contribute to the traffic operation benefit since this method requires a relatively shorter time compared to the conventional solution. In other words, the conventional method requires more than 4 h for the surface to cool to approximately 60 °C while the WGA only needs more than 3.5 h. Further investigating the thermal development in the warm guss mixture, when the thickness of the overlaying is 50 mm, the traffic open time based on 40 °C criteria is 4 h after overlaying, and this value of the 50 °C constraint is 2 h. Considering the overlaying thickness of 100 mm, the traffic opening time constrained on 40 °C is 6 h while the 50 °C condition requires nearly half that value of 3 h and 15 min after overlaying. Further investigation on the 150mm warm mix, the traffic opening time for the former and latter temperatures is 4 h and 5 min and 7 h and 40 min after overlaying, respectively. Especially, in view of the thickest overlaying layer of 250 mm, the allowable cooling time requires at least 10 h and 35 min and 5 h and 15 min for the 40 °C and 50 °C requirements, respectively.

Table 8 below summarizes the traffic opening times associated with the constrained temperatures of 40 °C and 50 °C. Based on the MOLIT [19] requirements for the opening of traffic, the surface temperature of 40 °C is a standard for covering a large area, and it is considered not to be suitable for the regulation of the fast installation of buried pipes in downtown areas. Moreover, the surface temperature during the opening of 50 °C is a more realistic standard for the mini-trench construction site. Findings confirm that WGA material can shorten the opening time from 30 min to 1 h and 25 min compared to HGA material based on an operating surface temperature of 50 °C. Through this, it is considered that the WGA mixture, which has lower production and laying temperature than the conventional HGA mixture, is superior in terms of traffic opening. In addition, when considering a situation where rapid traffic opening is required, such as a mini-trench construction in an urban area, it is expected that an overlaying depth of less than 100 mm is appropriate for practical construction purposes.

### 3.3. Temperature around Buried Pipes

The thermal analysis between warm and HGA overlaying was conducted to determine the cover thickness of the backfill concrete surrounding the buried pipe. In this research, the cover thickness (c) is the distance from the top of the outer diameter of the pipe to the top of the concrete backfill layer.

**Figure 9:**
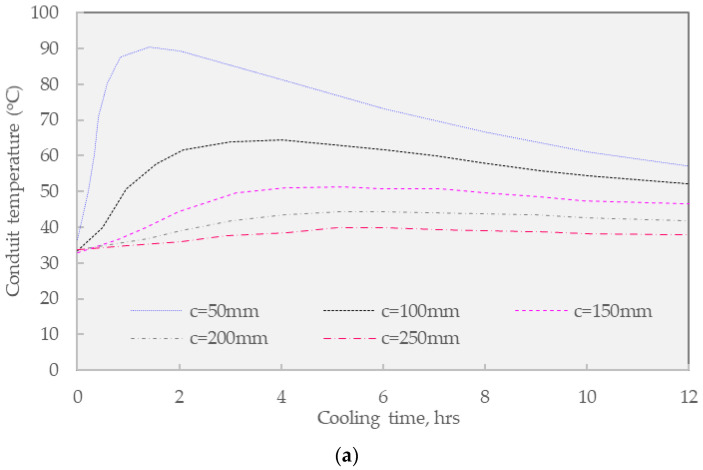

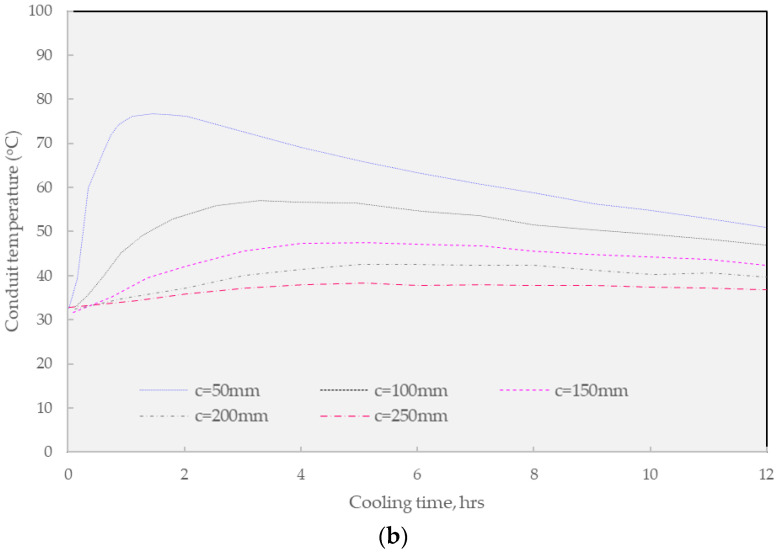
The temperature at the Top of the Conduit of FEA results. (**a**) Hot Mix Guss Mastic Asphalt; (**b**) Warm Mix Guss Mastic Asphalt.

The analysis in Figure 9 presents findings 12 h after overlaying construction, with the cover thickness of backfill concrete ranging from 50 mm to 250 mm under the fixed value of 100 mm overlaying guss asphalt. Figure 4 shows the analysis results for the temperature change at the top of the buried pipe. The maximum temperature of the buried pipe, according to KS C 8454, is shown together.

Figure 9a shows the impact of conventional HGA overlay on the designed covering thickness of backfill concrete. When the concrete cover is 250 mm, the maximum temperature at the top of the buried pipe rises to 39.6 °C, and it is confirmed that the maximum temperature used is within the standard of 60 °C [28]. Considering the backfill concrete cover of 150 mm, the measured temperature at this location was increased to 51.9 °C an acceptable value. However, when the concrete cover thickness is reduced to 100 mm, the maximum temperature at the top of the buried pipe rises to 64.1 °C which exceeds the maximum temperature limit of 60 °C for about 2 h and 20 min, thus this thickness option does not meet the standard. Finally, regarding the lowestconcrete cover thickness of 50 mm, the maximum temperature at the top of the buried pipe increased remarkably to 94.48 °C and surpassed the maximum temperature limit for a duration of about 9 h and 35 min, indicating the failure of this design. In general, the thickness of the concrete imposed a strong impact on the thermal value of the buried conduit.

Figure 9b presents the result of WGA overlaying on the designed thickness of backfill concrete for the conduit system. In general, the application of warm mix asphalt enhances the thermal durability of the buried conduit since this solution requires lower construction heat. Regarding the first, when the thickness concrete cover is 250 mm, the maximum temperature at the top of the buried pipe rises to 38.3 °C, which confirmed that the maximum temperature for use is within the standard that is lower than 60 °C. In addition to the largest thickness option, the selection of 100 mm covers thickness results in the maximum temperature at the top of the buried pipe of 57.1 °C which is very close to the standard. Finally, the shortest solution of 50 mm also suffered from the overwhelming temperature in the buried conduit since the measured temperature was 76.8 °C which last for about 7 h and 25 min.

**Figure 10:**
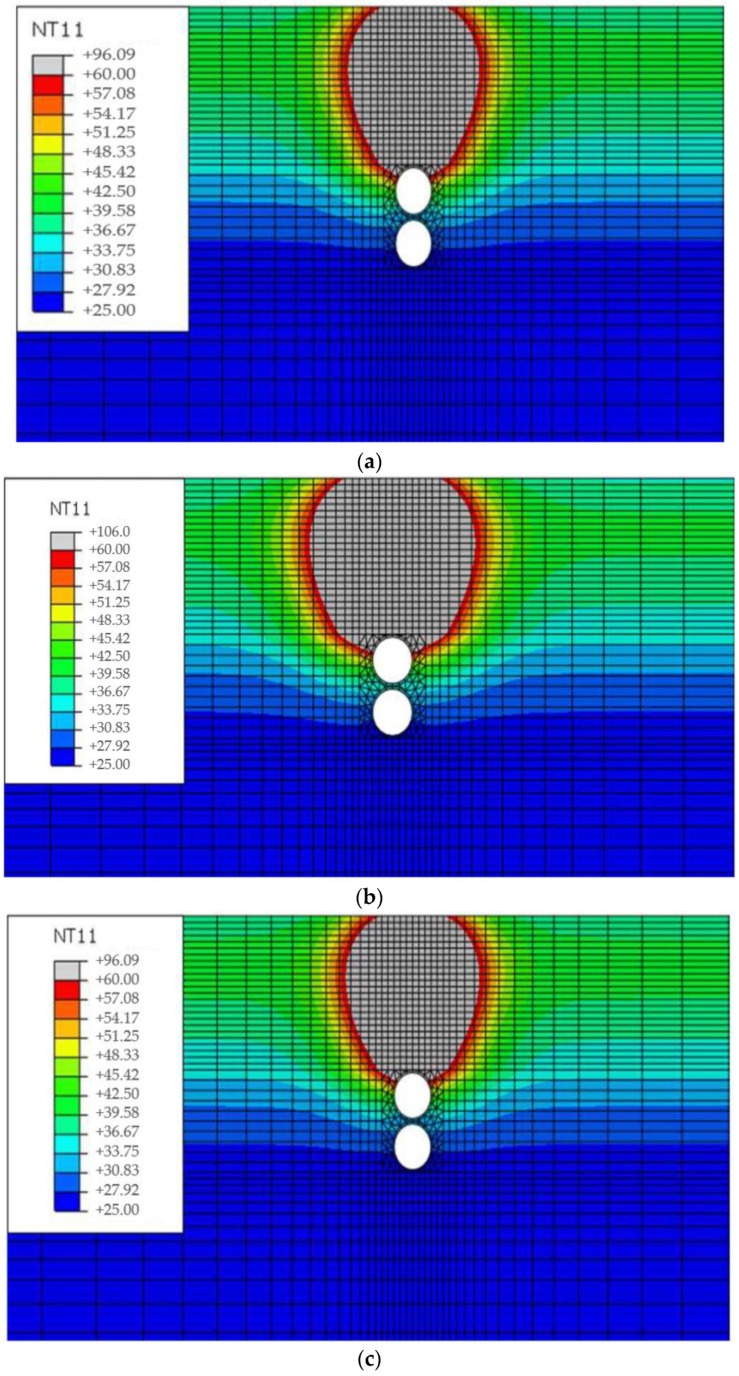
FE Analysis Results of Pavement Temperature Distribution. (**a**) Concrete Cover 50 mm (Warm Mix Guss Mastic Asphalt); (**b**) Concrete Cover 50 mm (Hot Mix Guss Mastic Asphalt); (**c**) Concrete Cover 100 mm (Hot Mix Guss Mastic Asphalt).

Following the findings in the above figures, Figure 10 shows the temperature distribution result of the pavement when the temperature of the buried pipe exceeds the KS C 8454 operating temperature limit [28], and the area exceeding 60 °C is displayed in gray color. Considering the WGA overlay as the heat source, the heat map shows that about 1/3 of the upper pipe circumference exceeded the standard when the backfill concrete cover thickness was designed at 50 mm. Especially under equivalent conditions, the conventional guss asphalt option indicates nearly 1/2 of the pipe circumferences could not meet the standard. This affected area was reduced by about to 1/3 when the concrete cover thickness was increased to 100 mm, noticing the essential design of concrete covering.

The following Table 9 summarizes the maximum temperature results at the top of the buried pipe following the KS C 8454 operating temperature standards [28]. It should be noted that the rigidity of the buried pipe for resin-based wires changes rapidly under the influence of temperature. Therefore, it is suggested to strictly follow the KS C 8454 operating temperature standard since the high-temperature effect on the wires in the buried pipe is very critical. The design of a proper thickness is essential for practical application. As regards the first, when the cover thickness of the backfilling concrete was 50 mm, not only the conventional HGA mixture but also the WGA mixture did not satisfy the criteria. Considering the conventional guss asphalt mixture, it was found that the concrete cover thickness of 100 mm could not meet the requirements, while this thickness of the warm mix asphalt concept meets the criterion.

Therefore, it was confirmed that the WGA mixture, which has a lower production and installment temperature than the conventional guss asphalt mixture, exhibits superior characteristics in terms of overlaying thickness, and the WGA mixture is suggested to be applied as a practical overlaying material. In general, it is suggested that the thickness of concrete cover should be designed higher than 100 mm to sustain the durability of buried conduit under guss mastic asphalt overlaying.

### 3.4. Verification of FEM Model

Additionally, in order to verify the Finite Element Method (FEM) model in the context of the research, the following steps were taken. The first stage of the research involved lab testing to determine the thermal conductivity and specific heat necessary for simulation work. To verify the FEM model, the accuracy of these lab results were confirmed through independent testing and analysis. The FEM model analysis was conducted based on the summer pavement temperature distribution through the Korean pavement Research Program. It is essential to ensure that the input data used in the FEM model accurately represents the conditions under which the mini-trench will be constructed and that the assumptions made in the model are reasonable. A sensitivity analysis was performed to determine how changes in the input parameters (asphalt thickness, concrete covering) affect the FEM model’s output as shown in the generated results. This analysis will help identify any potential sources of error or uncertainty in the model. However, the FEM model’s outputs should be further compared with actual measurements taken during and after construction to verify their accuracy. These measurements could include temperature readings of the pavement surface and buried conduit, as well as the time required for the asphalt mixture to cool to the required temperature for traffic opening.

**Figure 11:**
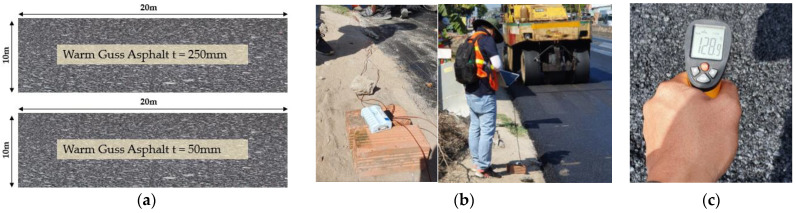
(**a**) Field testbed section, measurement of the WGA surface by using instrument (**b**) and thermal meter (**c**).

The field construction was performed in the South of Vietnam, in the Mekong delta region. The testbed section was designed in 40 m long sections involving 2 different conditions, WGA having a thickness of 250 mm and 50 mm, respectively. The ambient temperature was around 30 ± 3 °C on the testing day and the humidity is greater than 80%. Considering the mix design of the full-scale testbed, the pilot trenches were overlayed by the equivalent mix design used in the laboratory works. The measurement of the asphalt temperature in the field is presented in Figure 11. The following comparison was developed from the recent field test to verify the FEM modeling of the trench covered by WGA.

**Figure 12:**
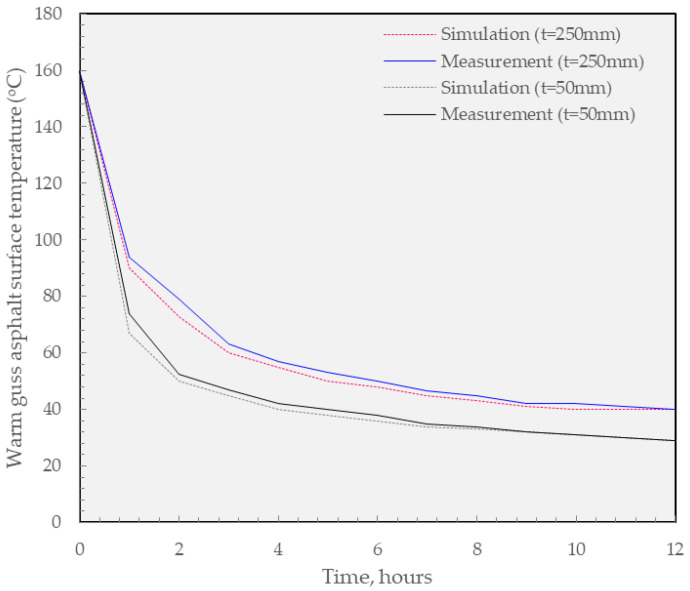
Comparison between the simulation and field measurement.

In general, as shown in Figure 12, the testbed measurement confirms that the general accuracy of the simulation works since both processes share the equivalent trend in temperature reduction through curing time. However, it should be noted that the measured section showed a slightly higher temperature compared to that of the simulation work. Especially, the greatest gap between them can be found between the initial 2 h. This can be explained by the fluctuation of temperature in the hot climate in South Vietnam. As a result, further calibration should be conducted to properly simulate the thermal regulation in the field.

## 4. Conclusions

The objective of this study was to perform a heat transfer analysis of an overlaying material containing warm guss asphalt (WGA) mixture for the mini-trench method in urban areas. The laboratory tests were initially conducted to extract the thermal conductivity of the proposed mixture. The thermal analysis was then performed by applying an installation temperature of 160 °C or 200 °C for WGA and the conventional hot guss asphalt (HGA) mixture, respectively. In addition, the asphalt mixtures thickness in this research varied from 50 mm to 250 mm to optimize the thickness investigation. The main conclusions are summarized as follows:

- Based on the laboratory test results, the thermal conductivity of the WGA mixture was relatively equivalent to the conventional mixture, and adding a proper content of Sasobit (1%) resulted in a low viscosity for warm application purposes.
- WGA was found to be superior in terms of traffic opening time compared to conventional HGA materials, as it could shorten the opening time from 30 min to 1 h and 25 min.
- The study found that the time required to open traffic increased as the depth of the WGA mixture overlay increased. Therefore, an overlaying depth of 100 mm was deemed reasonable for practical construction purposes, as it is difficult to conduct traffic blocks for more than 4 h at the site.
- The investigation of the minimum cover thickness of the backfill concrete that protects the buried pipe from the heat source from the above 100 mm guss asphalt layer found that WGA could be used to construct thinner backfill concrete surrounding the landfill pipe, resulting in a cost-effective effect. Additionally, it was found that a conventional HGA mixture must be designed with a concrete thickness of 150 mm to achieve proper covering purposes.
- The accuracy of the simulation is supported by the testbed measurement, as both processes exhibit a comparable trend in the reduction of temperature over time. Nevertheless, the temperature in the measured section was slightly higher than that of the simulated work, particularly during the initial two-hour period, potentially due to fluctuations in temperature in the hot climate of southern Vietnam.
- In summary, the study found that the WGA mixture is a viable option for the mini-trench method due to its low production and compaction temperatures and shorter traffic opening time. However, future research is needed to further evaluate the developed WGA mixture for practical construction purposes, and further adjustments are required to refine the simulation’s thermal regulation capabilities in the field.

## Figures and Tables

**Table 1:** Properties of guss mastic asphalt binder.

Properties	Value	Standard Value
Penetration (1/10 mm) 25 °C	87.2	
Softening point (°C)	68.6	
Ductility at 5 °C (cm/min)	105	
Thin film oven (160 °C, 300 min)		
Mass loss (%)	0.05	
Penetration loss	72	
G*/sinδ; at 76 °C (Original)	1.72 kPa	Min. 1.0 kPa
G*/sinδ at 76 °C (after RTFO)	2.41 kPa	Min. 2.2 kPa
G* × sinδ at 76 °C (after PAV)	1527 kPa	Max. 5000 kPa
Stiffness at −22 °C	186 MPa	Max. 300 MPa
m-value at −22 °C	0.32	Min. 0.3

**Table 2:** Aggregate and mineral filler properties.

Properties	Properties	Value
Aggregate	Relative apparent density	2.67
	Water absorption	0.18%
	Aggregate crushed value	19.5%
	Los Angeles abrasion value	25.8%
	Flakiness and elongation index	12.5%
Mineral Filler	Relative apparent density	2.36
	Moisture content	0.09%

**Table 3:** Sieve size gradation.

Sieve size (mm)	16	13.2	9.5	4.75	2.36	1.18	0.6	0.3	0.15	0.075
Gradation (%)	100	100	97.6	62.5	9.1	5.1	3.3	2.7	2.0	0.8

**Table 4:** General properties of guss mastic asphalt mixture (1% Sasobit).

Properties	Result Value	Standard Value
Stability (N)	11,789	≥5000
Flow value (1/100 cm)	29	20~40

**Table 5:** Overview of numerical analysis cases.

Warm Case	Content	Hot-Case	Content
50 mm–160 °C	Warm Guss mastic asphalt concrete & a depth of 50 mm	50 mm–200 °C	Hot Guss mastic asphalt concrete & a depth of 50 mm
100 mm–160 °C	Warm Guss mastic asphalt concrete & a depth of 100 mm	100 mm–200 °C	Hot Guss mastic asphalt concrete & a depth of 100 mm
150 mm–160 °C	Warm Guss mastic asphalt concrete & a depth of 150 mm	150 mm–200 °C	Hot Guss mastic asphalt concrete & a depth of 150 mm
200 mm–160 °C	Warm Guss mastic asphalt concrete & a depth of 200 mm	200 mm–200 °C	Hot Guss mastic asphalt concrete & a depth of 200 mm
250 mm–160 °C	Warm Guss mastic asphalt concrete & a depth of 250 mm	250 mm–200 °C	Hot Guss mastic asphalt concrete & a depth of 250 mm

**Table 6:** Properties of input materials used for simulation process.

Material	Properties	Value	Reference
Asphalt layer & Guss Mastic overlaying	Density, kg/m^3^	2373	Laboratory experiment results (Section 3.1) and Luca and Mrawira (2005) [31]
	Thermal Conductivity, W/mK	1872	
	Specific heat, J/kgK	1664	
Subgrade	Density, kg/m^3^	1975	Côté et al. (2005) [32] Ižvolt et al. (2014) [33,34]
	Thermal Conductivity, W/mK	2.5	
	Specific heat, J/kgK	1005	
Subbase	Density, kg/m^3^	1898	Kay et al. (1975) [36] Xu et al.(2020) [35]
	Thermal Conductivity, W/mK	1.5	
	Specific heat, J/kgK	1422	
Concrete backfills	Density, kg/m^3^	2300	ACI-122R-02 [37]
	Thermal Conductivity, W/mK	1.6	
	Specific heat, J/kgK	963	
PVC	Density, kg/m^3^	1380	
	Thermal Conductivity, W/mK	0.2	
	Specific heat, J/kgK	880	

**Table 7:** Temperature standard of synthetic resin flexible conduit.

Type	Min. Temp. for Storage and Transportation	Min. Temp. for Installation and Operation	Range of Operating Temp.
1	−5 °C	−5 °C	−5 °C~60 °C
2	−25 °C	−25 °C	−15 °C~60 °C

**Table 8:** Time required from construction to traffic opening.

Overlaying Depth	Traffic Opening Time			
	Warm Mix Guss Mastic Asphalt		Hot Mix Guss Mastic Asphalt	
	40 °C	50 °C	40 °C	50 °C
50 mm	4 h	2 h	4 h 40 min	2 h 30 min
100 mm	6 h	3 h 15 min	7h	4 h
150 mm	7 h 45 min	4 h 5 min	8 h 40 min	4 h 40 min
200 mm	9 h	4 h 55 min	10 h	6 h
250 mm	10 h 35 min	5 h 15 min	12 h 45 min	6 h 40 min

**Table 9:** FEA results in maximum temperature on the top of the conduit.

Concrete Cover	Warm Mix Guss Mastic Asphalt		Hot Mix Guss Mastic Asphalt	
	Max. Temp. (°C)	Operation Limit	Max. Temp. (°C)	Operation Limit
250 mm	38.3	Lower	39.6	Lower
200 mm	42.4	Lower	44.5	Lower
150 mm	48.0	Lower	51.9	Lower
100 mm	57.1	Lower	64.1	Higher
50 mm	76.8	Higher	94.8	Higher

## Data Availability

Data will be provided on request.
